# The Impact of a Twelve-Week Moderate Aerobic Exercise Program on Gastrointestinal Symptom Profile and Psychological Well-Being of Irritable Bowel Syndrome Patients: Preliminary Data from a Southern Italy Cohort

**DOI:** 10.3390/jcm12165359

**Published:** 2023-08-17

**Authors:** Giuseppe Riezzo, Laura Prospero, Benedetta D’Attoma, Antonia Ignazzi, Antonella Bianco, Isabella Franco, Ritanna Curci, Angelo Campanella, Caterina Bonfiglio, Alberto Ruben Osella, Francesco Russo

**Affiliations:** 1 Functional Gastrointestinal Disorders Research Group, National Institute of Gastroenterology IRCCS “S. de Bellis”, 70013 Castellana Grotte, Italy; giuseppe.riezzo@irccsdebellis.it (G.R.); laura.prospero@irccsdebellis.it (L.P.); benedetta.dattoma@irccsdebellis.it (B.D.); antonia.ignazzi@irccsdebellis.it (A.I.); 2 Laboratory of Epidemiology and Biostatistics, National Institute of Gastroenterology IRCCS “S. de Bellis”, 70013 Castellana Grotte, Italy; antonella.bianco@irccsdebellis.it (A.B.); isabella.franco@irccsdebellis.it (I.F.); ritanna.curci@irccsdebellis.it (R.C.); angelo.campanella@irccsdebellis.it (A.C.); catia.bonfiglio@irccsdebellis.it (C.B.); arosella@irccsdebellis.it (A.R.O.)

**Keywords:** abdominal pain, aerobic exercise, bloating, IBS, psychological profile, QoL

## Abstract

Walking is popular moderate-intensity aerobic exercise that improves mental and gastrointestinal (GI) health. It can relieve symptoms associated with irritable bowel syndrome (IBS), e.g., intestinal gas, abdominal distension, and bowel disturbances. This study examined the impact of a moderate-intensity aerobic exercise program on the clinical and psychological parameters of IBS patients. In total, 40 IBS patients (11 males and 29 females; mean age 51.9 ± 7.8 years) participated in a 12-week aerobic exercise program. Participants completed questionnaires assessing GI symptoms, psychological profiles, and quality of life (QoL) before and after the intervention. Field tests, anthropometric measurements, and bioimpedance assessments were also conducted. The present findings confirmed a significant improvement in IBS symptoms after the aerobic exercise program. Bloating was the most common symptom and, together with abdominal pain, was significantly reduced after treatment. Psychological and QoL questionnaires indicated decreased anxiety, depression, somatization, and stress levels. Correlations were found between anxiety/depression and the severity of abdominal pain as well as between stress and the severity of abdominal distension. Moderate-intensity aerobic exercise positively impacted GI symptoms and psychological well-being, complementing dietary and psychological support as a non-pharmacological therapy for the management of IBS. These findings emphasize the importance of alternative approaches for IBS treatment.

## 1. Introduction

Irritable bowel syndrome (IBS) is a chronic condition characterized by recurrent abdominal pain associated with bowel disturbances such as altered stool consistency and frequency [1]. Based on a literature review, there are different diagnostic criteria and consequent varying prevalence rates for this syndrome, ranging from 9% to 22% in Europe [2]. Patients suffering from IBS may experience a compromised quality of life (QoL) and incur significant personal and healthcare costs due to the burden of diagnostic tests and excessive medical treatments [3]. Several factors, including mental, physical, dietary, nutritional, and environmental, contribute to the etiology of IBS [4]. Pharmacological therapy has a well-defined role in addressing specific aspects of the syndrome. Still, it should only be used for short periods in conjunction with non-pharmacological strategies such as dietary approaches [5].

Our group recently published some studies on the effects of different diets, which share the exclusion or reduction of foods capable of inducing an osmotic effect and fermentation by intestinal bacterial flora in predisposed patients, leading to abdominal pain, bloating, and diarrhea, typical symptoms of IBS [6]. Specifically, diets such as the low FODMAP (fermentable oligo-saccharides, disaccharides, mono-saccharides, and polyols) diet and the alternative grain-based diet (e.g., tritordeum) have shown clear effects on symptoms, inflammation levels, and gastrointestinal (GI) permeability as well as on the psychological profile of IBS patients [7,8,9,10]. When supported by expert nutritionists, this approach can be used for an extended period, significantly affecting healthcare costs [11].

Another interesting approach is based on well-defined physical activity (PA) programs. The World Health Organization guidelines on PA and sedentary behavior (2020) state that healthy adults should engage in regular activity consisting of at least 150–300 min per week of moderate-intensity aerobic exercise or at least 75–150 min per week of vigorous-intensity aerobic activity or a combination of both to achieve noticeable health benefits [12].

PA can improve cardiovascular parameters and GI variables such as excess lower intestinal gas, abdominal distension, and bowel disturbances, which are key symptoms in the profile of IBS patients [13]. Additionally, conditions like depression and fibromyalgia, often associated with IBS, seem to benefit from regular exercise [14,15]. Research has repeatedly proved that regular exercise can improve mental health and significantly reduce symptoms of depression, anxiety, and generalized stress [16]. In this regard, some studies have suggested that PA can enhance mental well-being as much as psychotherapy [17].

Case-control studies revealed that IBS patients engaged in less PA than non-IBS controls [18]. The recommended average PA for IBS patients consists of 30–60 min of moderate-intensity exercise 3 to 5 times a week for at least 12 weeks [19]. The most common form of PA is walking exercise, typically used with other PA protocols, which can affect IBS symptoms [20].

Despite these premises, while numerous authors have highlighted the evident beneficial PA impact on IBS symptoms and QoL of patients in different parts of the world, to our knowledge, little—if any—information is available on the effect of PA in a cohort of IBS patients from South Italy. To confirm this finding, we can only report a paper on healthy subjects from South Italy highlighting the link between GI symptoms and reduced PA due to COVID-19 home confinement. This context is undoubtedly different from the one described above [21].

On these bases, the main goal of the present study was to examine the impact of a 12-week moderate aerobic exercise program from a multidimensional perspective, assessing the GI symptomatic and psychological profile along with the anthropometric status in a cohort of IBS patients from South Italy.

## 2. Materials and Methods

### 2.1. Patient Recruitment

Patients suffering from IBS in accordance with Rome III–IV criteria [17] were recruited from July 2022 to May 2023 from the outpatients of the Functional Gastrointestinal Disorders Unit, National Institute of Gastroenterology, IRCCS “S. de Bellis”.

As preliminary screening, the Gastrointestinal Symptom Rating Scale (GSRS) questionnaire [22] was issued during the consultations. Patients (18–65 years) had to report IBS-like symptoms ranging from mild to moderate according to the Irritable Bowel Syndrome Severity Scoring System (IBS-SSS) (see Section 2.4.1) that had been present for at least two weeks.

All patients required blood tests to have been completed within the past three months for liver and thyroid functions, C-reactive protein, FOBTs from three determinations, stool cultures, and stool tests for parasites. A recent gastroscopy and colonoscopy with biopsy samples were also required.

Criteria of exclusion were pregnancy; a diagnosis of metabolic or endocrine disorders; hepatic, renal, cardiovascular, neurological, or psychiatric disease; constipation; post-infectious IBS; giardiasis; prior abdominal surgery; fever; vigorous exercise; secondary causes of intestinal atrophy; a history of malignancy; probiotic therapy; use of selective serotonin reuptake inhibitors; and no therapies for IBS in the last two weeks before evaluation. Tissue transglutaminase and anti-endomysium antibodies were analyzed as celiac markers. Patients could not consume overly nutrient-restrictive diets before enrolling in the trial (such as gluten-free, vegan, or low FODMAP diets).

The case report form contained a list of the justifications for leaving the study, including adverse occurrences (described), ineligibility to continue the study, withdrawal of permission, lost to follow-up, and other causes (described).

The local scientific committee and the Institutional Ethics Committee of the IRCCS Oncological Hospital “Giovanni Paolo II Cancer Institute”, Bari, Italy, approved the present study (Prot. No. 177/EC, 13 May 2022; registered at clinicaltrial.gov as NCT05453084, last access date 12 July 2022).

### 2.2. Study Design

**Figure 1:**
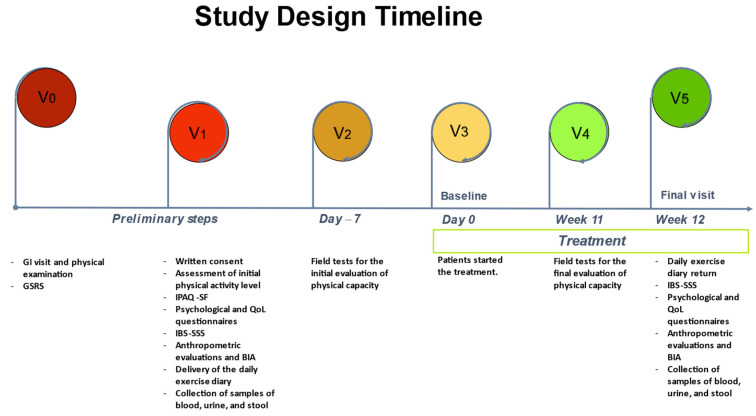
The study design timeline. GI: gastrointestinal; GSRS: Gastrointestinal Symptom Rating Scale; IPAQ-SF: International Physical Activity Level Questionnaire; QoL: quality of life; IBS-SSS: Irritable Bowel Syndrome Symptom Severity Scale; BIA: bioelectrical impedance analysis.

The study design timeline (Figure 1) was organized as follows:

V_0_: All patients underwent GI visits and physical examinations and completed the GSRS questionnaire.

V_1_: Patients were cooperative and ready to participate, received information about the study’s goal, and signed an informed consent form. Information on their level of physical activity and any issues or illnesses was collected to determine whether they were fit enough for the intended workout. Qualified personnel (graduates in science and techniques of preventive and adapted motor activities) also administered the International Physical Activity Questionnaire (IPAQ-SF) and provided instructions to correctly complete the daily exercise diary. During the session, patients also filled out psychological questionnaires such as the Symptom Checklist-90-Revised (SCL-90-R) and Psychophysiological Questionnaire (QPF/R) as well as QoL questionnaires such as the Irritable Bowel Syndrome Quality of Life (IBS-QoL) and Thirty-Six-Item Short-Form Health Survey (SF-36), in addition to GI-symptom questionnaires such as the Irritable Bowel Syndrome Severity Scoring System (IBS-SSS). On the same day, patients underwent a bioelectrical impedance analysis (BIA) and the anthropometric evaluations (weight, height, and circumferences) were performed by a trained nutritionist. Lastly, samples of blood, urine, and stool were collected.

V_2_: No more than 7 days after V_1_ and, in any case, 7 days before the beginning of the exercise intervention, the enrolled subjects, after presenting a certificate of physical fitness, underwent three field tests for the initial evaluation of physical capacity.

V_3_: Patients started the treatment.

V_4_: In the seven days before the end of the aerobic exercise program, the patients performed the three physical field tests again, as in V_2_.

V_5_: Patients handed in their daily exercise diaries and completed the GI-symptom questionnaire (IBS-SSS). Psychological and QoL questionnaires were also administered. An anthropometric assessment and BIA were carried out and potential adverse events during the intervention were assessed. Finally, a blood sample and biological samples were taken.

### 2.3. Anthropometric and Bioelectrical Impedance Analysis (BIA) Parameters

The following anthropometric parameters were evaluated: height, weight, body mass index (BMI), mid-upper arm, waist, and hip circumference. A SECA 700 mechanical column scale and a SECA 220 altimeter (INTERMED S.r.l., Milan, Italy) were used to assess the subjects’ weight and height to calculate their BMI (kg/m^2^).

All patients who underwent the BIA had fasted for at least 4 h and abstained from alcohol and strenuous exercise for the preceding 12 h.

Through the injection of a continuous (800 A) sinusoidal current at a frequency of 50 kHz, a BIA measures the resistance (Rz) and reactance (Xc) of human tissue. The same instrument (BIA 101 BIVA PRO, Akern SRL, Pontassieve, FI, Italy) was used for all measurements, which were carried out under exacting standards in accordance with the recommendations of the European Society for Parenteral and Enteral Nutrition [23]. Body cell mass, fat-free mass, fat mass, total body water, and extracellular water were calculated from the Rz and Xc using specialized software (Bodygram PLUS Software v. 1.0, Akern SRL, Pontassieve, FI, Italy). The phase angle (calculated as the arctangent of the Xc/Rz ratio) was also calculated.

### 2.4. Gastrointestinal Questionnaires

#### 2.4.1. Irritable Bowel Syndrome Severity Scoring System (IBS-SSS)

The IBS-SSS was administered to evaluate the GI-symptom profile. Five categories make up this validated questionnaire, including “severity of abdominal pain”; “frequency of abdominal pain”; “severity of abdominal bloating”; “dissatisfaction with bowel habit”; and “interference with life in general”, with a maximum total score of 500. The severity of IBS was determined using the generally accepted IBS-SSS cut-off scores of >75–175 for mild IBS, 175–300 for moderate IBS, and >300 for severe IBS [24].

### 2.5. Psychological Questionnaires

#### 2.5.1. Irritable Bowel Syndrome Quality of Life Questionnaire (IBS-QoL)

The IBS-QoL is a self-reported QoL measure specific to IBS and is used to assess the impact of IBS and its treatment. It consists of 34 items summed and averaged for a total score. There are also eight subscales: dysphoria, interference with activity, body image, health worry, food avoidance, social reaction, sexual, and relationships. Higher ratings indicate a higher QoL. Raw results are translated into scale scores ranging from 0 to 100 [25].

#### 2.5.2. Thirty-Six-Item Short-Form Health Survey (SF-36)

The SF-36 is a brief survey designed to examine patients’ QoL and assess their overall health, regardless of the sickness they have complained about. The global and subscale indices are set up so that a higher score indicates a better state of health. The first three subscales measure physical activity, limit role-specific actions brought on by physical issues and pain, and define physical health. The two intermediate subscales (general health and vitality) describe the overall state of global health. The final three subscales concern the elements of psychological and emotional well-being (restrictions on social interactions and limitations on role-specific activities brought on by emotional or mental health issues). An additional unscaled single item provides information on the respondent’s health changes over the previous year. It is also possible to calculate the values of two synthetic indices, one relating to physical health (physical health scale) and the second to mental health (mental health scale). These indices are derived from the eight scales and make it possible to summarize the results of all scales in just two scales. Scores are coded, added up, and transformed for each variable on a scale from 0 (the worst possible health state) to 100 (the highest possible health state) [26].

#### 2.5.3. Symptom Checklist-90-Revised (SCL-90-R)

The SCL-90-R is a commonly applied tool for self-reporting psychological symptoms [27]. With nine core symptom dimensions (somatization, obsessive-compulsive, interpersonal sensitivity, depression, anxiety, hostility, phobic anxiety, paranoid ideation, and psychoticism) and three global indexes, the SCL-90-R assesses a broad spectrum of psychopathological symptoms. The present study adopted the Global Severity Index (GSI) because it accurately captured the degree of psychological anguish that the participants experienced. A T score equal to or greater than 63 is regarded as a clinically relevant symptom once the raw value has been transformed.

#### 2.5.4. Psychophysiological Questionnaire (QPF/R)

The QPF/R is included in Cognitive Behavioral Assessment 2.0 (Giunti psychometrics, Firenze, Italy), a test battery that offers a broad overview of psychological issues in individual and social domains. It consists of ten schedules; the sixth contains the QPF/R, which was used in the present study to evaluate the stress and psychophysiological alterations [28].

### 2.6. Evaluation of Physical Capacity

#### 2.6.1. Field Tests

The field tests used to assess physical capacity were a 2 km walking test (which evaluates the cardio-respiratory capacity), a hand grip test (which sets the maximum isometric strength of the forearm muscles), and the sit and reach test. This test evaluates the flexibility of the muscles of the lower back and the back of the thigh.

#### 2.6.2. Characteristics of the Exercise Program

The proposed exercise intervention was moderate aerobic exercise supervised by trained staff and carried out through a “walking group”. This term defines an organized and collectively conducted motor activity with the primary objective of promoting physical activity and improving quality of life. Walking group participants meet regularly at a predefined location to walk together along urban and suburban routes, guided and supervised by an experienced walking leader. Participation in a walking group is voluntary and free of charge for participants in research projects at our Institute.

Walking exercise allows training endurance, strength, speed, flexibility, and coordination, albeit at different intensities and times [29]. The precise walking technique was explained to the subjects before starting the exercise program; this allowed the participants to walk well and faster, thus producing positive metabolic effects through improved physical efficiency.

Our effective prescription included a systematically designed and individualized exercise program regarding different parameters such as frequency, intensity, type, time, and volume.

- Frequency: walking exercise was carried out outdoors on an urban route three times a week on non-consecutive days for 12 weeks.
- Intensity: the intensity of the exercise was 60–75% of HRmax; it was monitored using a heart-rate monitor and customized using Tanaka’s formula [30]. In addition, a talk test (a standardized and validated survey instrument based on the exercise subject’s ability to hold a conversation) was used to measure pace [31] and a modified Borg scale was used to measure the perception of fatigue (on a scale of 0–10, a perception of 5–6 was required) [32].
- Type: the type of exercise was moderate aerobic, with speeds ranging from 5 to 10 km/h.
- Time: A single outing, lasting 60′, was structured as follows: warm-up, 5′; normal walk, 10′; sustained walking, 30′; fast walking, 10′; cool-down, 5′.
- Volume: participants in the project performed 180 min per week of moderate-intensity aerobic exercise, following indications from the American College of Sports Medicine (ACSM) guidance on preventive health [33]. However, participants could also increase their exercise volume through activities outside the project. All exercise data within and outside the project were recorded in the daily diary to evaluate energy expenditure.

Patients were not allowed to be absent more than 20% of the time and adherence to the exercise program was assessed based on the percentage of sessions in which the subject was present and the percentage of sessions in which the intended intensity was achieved.

### 2.7. Statistical Analysis

Data were analyzed according to a per-protocol analysis; this indicated that patients reached the study endpoint without dropping out and completed all key assessments at the study visits, showing good treatment adherence. All results were presented as means ± SEM unless otherwise stated. The Wilcoxon matched-pairs signed-rank test, a non-parametric test, was performed to compare pre- and post-walking exercise because of the number of patients studied and to avoid the assumption of a normal distribution. The putative relationships between symptoms, anthropometric measurements, and psychological scales were examined using the Spearman correlation analysis. The statistical packages used were Sigma Stat 11.0 (Systat Software, Inc., San Jose, CA, USA) and GraphPad Prism 5 (GraphPad Software, Inc., La Jolla, CA, USA). A *p*-value less than 0.05 was considered to be statistically significant.

## 3. Results

### 3.1. Patients’ Demographic, Anthropometric, and Bioelectrical Impedance Characteristics

A total of 40 IBS patients (29 F; 11 M) were recruited. The overall mean age was 51.9 ± 7.8 years. Table 1 describes the IBS patients’ anthropometric and BIA characteristics before and after the aerobic exercise program. All anthropometric measures decreased and some significantly reduced, such as mid-upper arm and hip circumferences. Regarding BIA parameters, there were no apparent changes.

### 3.2. Gastrointestinal (GI) Symptoms

All scores obtained from the IBS-SSS were significantly reduced at the end of the intervention (Table 2). At the baseline, patients had an average IBS-SSS total score equal to 183.1 ± 12.56, which was classified as moderate, whereas after the physical exercise intervention, it dropped to a mild level (111.8 ± 12.15). The percentage of total score reduction was 38.9%. Regarding the individual items, the dominant symptoms at the baseline were “severity of abdominal bloating” and “dissatisfaction with bowel habit”. The item showing the most significant reduction percentage after treatment was “frequency of abdominal pain” (60.4%), while “severity of abdominal pain” decreased by 48.6%. The reduction in the “interference with life in general” score was less marked (27.4%), although still significant. Finally, positive correlations were found between “dissatisfaction with bowel habit” and “interference with life in general” (r = 0.45; *p* < 0.0001)

### 3.3. Psychological and Quality of Life Profiles

**Figure 2:**
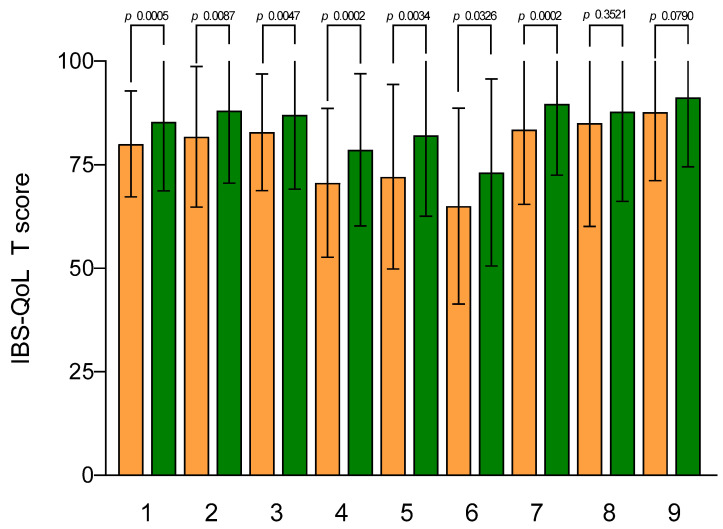
IBS-QoL subscale scores before and after aerobic exercise. Data are expressed as means ± SEM and were analyzed by the Wilcoxon matched-pairs signed-rank test. All differences were considered to be significant at *p* < 0.05. Subscales: (1) total score; (2) dysphoria; (3) interference with activity; (4) body image; (5) health worry; (6) food avoidance; (7) social reaction; (8) sexual; (9) relationships. Orange columns: pre; green columns: post.

The mean IBS-QoL total and subscales scores at the baseline and after walking exercise are shown in Figure 2. The IBS-QoL mean total score at the baseline was 80.02 ± 2.02 and significantly increased after the aerobic exercise program (85.34 ± 2.63; *p* = 0.0005). Regarding IBS-QoL subscales, there was a statistically significant increase after aerobic exercise for all subscales, except for the sexual one.

**Figure 3:**
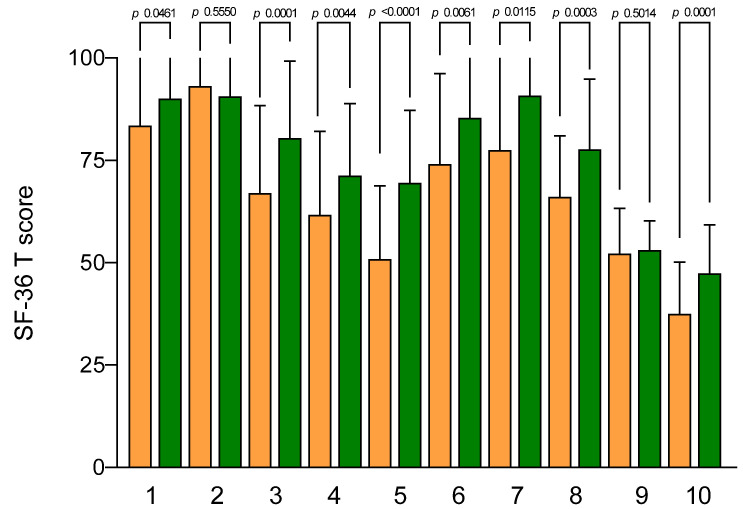
SF-36 subscale scores before and after aerobic exercise. Data are expressed as means ± SEM and were analyzed by the Wilcoxon matched-pairs signed-rank test. All differences were considered to be significant at *p* < 0.05. Subscales: (1) physical function; (2) physical role; (3) body pain; (4) general health; (5) vitality; (6) social functioning; (7) role emotional; (8) mental health; (9) physical health scale; (10) mental health scale. Orange columns: pre; green columns: post.

Figure 3 describes the mean scores of the SF-36 subscales. At the baseline, the lowest SF-36 scores were for the mental health scale (37.45 ± 2.00), vitality (50.88 ± 2.83), and physical health scale (52.23 ± 1.74), while the highest scores were for role limitations due to physical problems (physical role) and physical function (93.13 ± 6.99 and 83.50 ± 4.40, respectively).

After the aerobic exercise program, all subscales had a statistically significant improvement, except for role limitations due to physical problems (physical role) and physical health scale.

**Figure 4:**
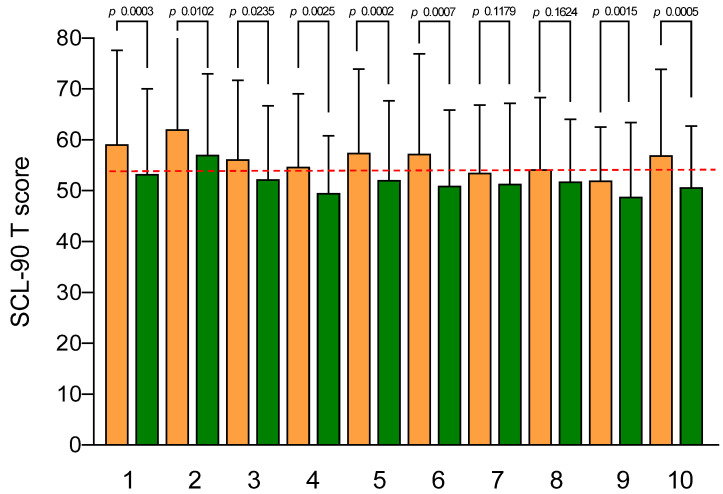
SCL-90-R subscale scores before and after the aerobic exercise. Data are expressed as means ± SEM and were analyzed by the Wilcoxon matched-pairs signed-rank test. All differences were considered to be significant at *p* < 0.05. Subscales: (1) GSI; (2) somatization; (3) obsessive-compulsive; (4) interpersonal sensitivity; (5) depression; (6) anxiety; (7) hostility; (8) phobic anxiety; (9) paranoid ideation; (10) psychoticism. The dotted red line indicates the cut-off score (63). Orange columns: pre; green columns: post.

The SCL-90-R results are illustrated in Figure 4. At the baseline, no subscale exceeded the cut-off value (63), even if GSI and somatization were in the upper limits (59.13 ± 18.48 and 62.08 ± 3.01, respectively). After the aerobic exercise, all SCL-90-R subscale scores significantly decreased, except for hostility and phobic anxiety. A significant correlation was found between the GSI, anxiety, and depression scores and “severity of abdominal pain” (r = 0.420, r = 0.408, and r = 0.390, respectively; *p* < 0.0001).

Finally, the level of stress experienced by the patients was assessed (QPF/R). The psychophysiological activation at the baseline was particularly high (62.83 ± 2.01; cut-off, 50). After the aerobic exercise program, the score statistically significantly decreased, despite persisting above the cut-off levels (55.25 ± 1.64; *p* < 0.0001). A significant correlation was found between the stress level and “severity of abdominal distension” (r = 0.379; *p* = 0.0005).

## 4. Discussion

The findings of the present study revealed a significant improvement in IBS symptoms following a 12-week moderate-intensity aerobic exercise intervention. The most prevalent symptom observed was bloating rather than abdominal pain and both of them were significantly reduced after the treatment. Moreover, various items in the psychological and QoL questionnaires demonstrated the positive impact of aerobic exercise.

IBS is associated with physiological and psychological issues, with substantial social costs [1]. The existing literature has primarily focused on various etiopathogenic factors contributing to IBS such as alterations in intestinal motility, GI peptides, mucosal modifications, and intestinal bacterial flora [2]. Different therapeutic approaches have been proposed based on the predominant etiopathogenic hypothesis [3,4,5]. While medications play a role in the initial phase, dietary modifications have been reported to provide greater long-term satisfaction, as highlighted in our previous studies [7,8,9,10].

A sedentary lifestyle and low PA as etiological agents contributing to IBS have received limited attention. The mechanisms underlying the association between PA and IBS remain unknown. PA can positively influence brain plasticity by facilitating neurogenerative, neuroadaptive, and neuroprotective processes [34], positively affecting the brain–gut axis involved in IBS. PA has been shown to reduce abdominal pain, help manage gas and colon transit [13], facilitate bowel movements [35], and alleviate anxiety [36]. A recent meta-analysis [37] suggested that different types of supervised and unsupervised PA could improve IBS symptoms, although the evidence level was low and data regarding adverse events were inconclusive. Therefore, high-quality studies, including randomized controlled trials, are required to draw definitive conclusions. Walking is one of the most popular forms of moderate-intensity PA due to its accessibility and minimal requirements. It offers a wide range of cardiovascular and GI health benefits [38,39,40,41].

Our study assessed the effects of moderate-intensity aerobic exercise on IBS symptoms using the IBS-SSS questionnaire. This questionnaire is commonly used to determine the severity and frequency of GI symptoms and to evaluate their interference with daily life [9]. Other studies demonstrated that interventions involving running, jogging, cycling, and swimming significantly reduced the total IBS-SSS score compared with respective control groups [20,38]. A cohort study on runners and walkers found that moderate-intensity PA such as walking provided similar benefits to high-intensity running in reducing the risk of hypertension, hypercholesterolemia, and diabetes after six years of follow-ups [38]. Our study confirmed a reduction in symptoms after aerobic exercise, as indicated by changes in the total and individual item scores of the IBS-SSS. At the end of the treatment, the total score changed from moderate to mild, representing a 38.9% reduction. It is worth noting that GI symptoms may hinder IBS patients, despite their motivation to engage in PA [42,43], underscoring the importance of selecting patients with mild to moderate symptoms to ensure better adherence to the activity.

An exciting finding pertained to the dominant symptom observed at the beginning of the study, which was abdominal bloating rather than abdominal pain, contrary to the characterization of IBS based on the Rome criteria [1]. This observation aligned with previous studies on the dominant symptom in female IBS patients subjected to a specific diet [44]. However, the most significant percentage reduction, with a value of 60%, was observed in the frequency of abdominal pain, indicating an effect on this particular symptom.

Regarding anthropometric characteristics, we observed a slight reduction in BMI—borderline significant—and a noticeable decrease in the mid-upper arm and hip circumferences. This finding may be attributed to the lack of dietary advice in the study design and was consistent with the previous literature on the effect of aerobic exercise on obese patients [39]. To achieve a sustainable weight reduction, it is crucial to incorporate lifestyle changes alongside PA.

PA has been demonstrated to alleviate somatic symptoms and impact stress [45], anxiety [46], depression [47], and overall emotional well-being [48]. Sports activities can help reduce anxiety and stress by distracting individuals from worries and releasing emotional tension. Regarding the QoL assessment, our patients had significantly improved total IBS-QoL scores and individual items related to dysphoria, interference with activity, body image, health worry, food avoidance, and social reaction. Psychological questionnaires revealed improvements in parameters such as anxiety, depression, and somatization, which are well-recognized disorders associated with IBS [11]. Moreover, the mental health scale of the SF-36 questionnaire significantly improved after the aerobic exercise period.

An interesting topic for discussion would be understanding the relationship between GI symptoms and the psychological profile induced by PA in general and aerobic exercise in particular. It remains unclear to what extent symptom improvement leads to psychological improvement or vice versa, or whether PA simultaneously affects both aspects. The psycho-biological model has been suggested as the most probable explanation. However, the specific role of basic mechanisms such as inflammation, oxidative stress, endocrine system, self-esteem, self-efficacy, and social support requires further clarification [14].

In our study, the “severity of abdominal pain” score correlated with the GSI, anxiety, and depression subscales of the SCL-90-R. On the other hand, “severity of abdominal bloating” correlated with the QPF/R, indicating the necessity of examining IBS patients from both gastroenterological and psychological perspectives, considering the dual etiology of the syndrome [11].

This research had some limitations. Firstly, it was an uncontrolled intervention study, lacking the robust evidence strength of a randomized controlled trial. The placebo effect of treatment should be considered in patients with IBS because it is higher (20–40%) than in other diseases [49]. It essentially concerns subjective responses to treatment, even when these actions are structured in validated questionnaires. In the present study, the anthropometric variables, which were certainly not influenced by a placebo effect, significantly decreased, such as mid-upper arm and hip circumferences [50]. Additionally, the inclusion criteria only encompassed patients with mild to moderate scores of IBS-SSS without distinguishing patients according to their bowel characteristics. Lastly, no biochemical evaluations were conducted, although they may be performed for future publications.

## 5. Conclusions

Utilizing alternative non-pharmacological therapies is a reasonable strategy for IBS. Both dietary adjustments and regular moderate-intensity aerobic exercise alongside essential psychological support can enhance GI symptoms and psychological well-being in equal measure. However, a critical inquiry emerges as to whether these approaches are synergistic or mutually exclusive, contingent upon the patient’s characteristics, symptom severity, bowel-movement patterns, and psychological profile. Exploring this aspect further could serve as a fruitful avenue for future research.

## Figures and Tables

**Table 1:** IBS patients’ anthropometric and bioelectrical impedance characteristics before (pre) and after (post) aerobic exercise program.

	Pre	Post	*p*-Value
Height (m)	164.2 ± 1.50	//	//
Weight (kg)	78.5 ± 2.49	77.9 ± 2.57	0.0847
BMI (kg/m^2^)	29.0 ± 0.81	28.8 ± 0.81	0.0580
Mid-upper arm circumference (cm)	33.6 ± 0.60	32.9 ± 0.61	0.0002
Waist circumference (cm)	93.0 ± 2.12	92.8 ± 2.16	0.2204
Hip circumference (cm)	106.3 ± 1.59	105.6 ± 1.58	0.0474
PhA (degrees)	6.4 ± 0.17	6.4 ± 0.15	0.9284
BCM (kg)	29.1 ± 1.11	28.9 ± 1.06	0.6538
FM (kg)	27.4 ± 1.70	27 ± 1.72	0.2200
FFM (kg)	52.0 ± 1.48	51.5 ± 1.43	0.1206
TBW (liters)	37.8 ± 1.08	37.7 ± 1.04	0.1976
ECW (liters)	16.6 ± 0.43	16.5 ± 0.41	0.6436

BMI: body mass index; PhA: phase angle; BCM: body cell mass; FM: fat mass; FFM: fat-free mass; TBW: total body water; ECW: extracellular water. Data are expressed as means ± SEM and were analyzed by the Wilcoxon matched-pairs signed-rank test. All differences were considered to be significant at *p* < 0.05.

**Table 2:** IBS-SSS single items and total score before (pre) and after (post) aerobic exercise program.

Item	Pre	Post	% Reduction	*p*-Value
Severity of abdominal pain	24.5 ± 4.31	12.6 ± 3.46	48.6%	0.0001
Frequency of abdominal pain	21.7 ± 4.72	8.6 ± 2.87	60.4%	0.0003
Severity of abdominal bloating	45.7 ± 3.79	27.8 ± 3.38	39.2%	0.0001
Dissatisfaction with bowel habit	47.7 ± 5.09	31.2 ± 3.78	34.6%	0.0002
Interference with life in general	43.4 ± 4.06	31.5 ± 4.30	27.4%	0.0077
Total score	183.1 ± 12.56	111.8 ± 12.15	38.9%	0.0001

Data are expressed as means ± SEM and were analyzed by the Wilcoxon matched-pairs signed-rank test. All differences were considered to be significant at *p* < 0.05.

## Data Availability

The datasets used and/or analyzed during the current study are available from the corresponding author upon reasonable request.
